# Efficacy and safety of nitazoxanide and escitalopram as adjuvant therapies in patients with rheumatoid arthritis: a randomized controlled study

**DOI:** 10.1007/s00228-025-03911-w

**Published:** 2025-09-09

**Authors:** Tarek M. Mostafa, Abeer A. El-Sayed, Abdel Moaty A. Afifi, Dalia R. El-Afify

**Affiliations:** 1 Department of Clinical Pharmacy, Faculty of Pharmacy, Tanta University, Tanta, Egypt; 2 Department of Pharmacy Practice, Faculty of Pharmacy, Arish Branch, Sinai University, Arish, Egypt; 3 Department of Physical Medicine, Faculty of Medicine, Rheumatology & Rehabilitation, Mansoura University, Mansoura, Egypt

**Keywords:** Rheumatoid arthritis, Nitazoxanide, Escitalopram, STAT-3, JAK-2, TLR-4, IL-1β

## Abstract

**Objective:**

This research aimed at evaluating the effectiveness and safety of nitazoxanide and escitalopram as adjuvant therapies in patients with rheumatoid arthritis (RA).

**Methods:**

In this randomized controlled parallel study, 90 patients with active RA were randomized into three groups; group 1 (control group; *n* = 30) which received traditional therapy, group 2 (Nitazoxanide group; *n* = 30) which received traditional therapy plus 1 gm/day oral nitazoxanide, and group 3 (Escitalopram group; *n* = 30) which received traditional therapy plus 10 mg/day oral escitalopram for three months. At baseline and 3 months after treatment, clinical and functional assessments were done through the 28-joint count disease activity score using C-reactive protein (DAS28-CRP), the health assessment questionnaire-disability index (HAQ-DI), and the patient’s global assessment (PGA). Also, serum levels of high-sensitivity C-reactive protein (hs-CRP), signal transducer and activator of transcription-3 (STAT-3), Janus kinase-2 (JAK-2), toll-like receptors 4 (TLR-4), interleukin-1 beta (IL-1β), and malondialdehyde (MDA) were assessed. Data were analyzed using paired *t*-test and one-way analysis of variance, followed by Tukey’s HDS test.

**Results:**

Three months after treatment and as compared to the control group, the nitazoxanide group showed a significant decline in PGA (*P* = 0.042), and serum levels of STAT-3 (*P* < 0.001), JAK-2 (*P* < 0.001), TLR-4 (*P* < 0.001), and IL-1β (*P* < 0.001). On the other hand, the escitalopram group produced a significant decrease in DAS28-CRP score (*P* = 0.029), HAQ-DI score (*P* = 0.001), and serum levels of JAK-2 (*P* = 0.001), TLR-4 (*P* < 0.001), IL-1β (*P* < 0.001), and MDA (*P* < 0.001). As compared to nitazoxanide group, the escitalopram group produced a significant decline in fatigue score (*P* < 0.001) and serum levels of both IL-1β (*P* = 0.023) and MDA (*P* < 0.001). Both medications were safe; however, chromaturia was the only significant nitazoxanide-related adverse effect.

**Conclusion:**

Nitazoxanide and escitalopram could serve as potential adjuvant therapies for patients with RA based on their effectiveness and safety data.

## Introduction

Rheumatoid arthritis (RA) is a chronic autoimmune disease affecting approximately 1% of the global population [1, 2]. In Egypt, patients with RA often experience higher disease activity and increased comorbidity rates, which are partially attributed to limited access to advanced therapies due to economic barriers and insufficient governmental or insurance support [3].

Angiogenesis plays a significant role in the pathogenesis of RA [4]. The signal transducer and activator of transcription-3/Janus kinase-2 (STAT-3/JAK-2) signaling pathway regulates angiogenesis via modulation of vascular endothelial growth factor (VEGF) expression [5–8]. Preclinical studies demonstrated that inhibition of STAT-3 exerts significant anti-inflammatory effects in models of colitis [9], sepsis [10], and arthritis [11]. Therefore, targeting STAT-3/JAK-2 could be effective for treating RA.

Toll-like receptor-4 (TLR-4) also contributes to progression of RA via promoting inflammation-driven angiogenesis through certain cytokines such as tumor necrosis factor–alpha (TNF-α), interleukin-6 (IL-6), and interleukin-1 beta (IL-1β) [12, 13]. Interleukin-1 beta (IL-1β) plays a crucial role in the inflammatory response and is involved in a wide range of cellular functions, including cell proliferation, differentiation, and programmed cell death [14]. Therefore, TLR4/IL-1β signaling might be a promising target for treating RA.

Additionally, oxidative stress is common during RA and could promote angiogenesis through enhancing VEGF expression and VEGFR2 activation [15, 16]. Moreover, reactive oxygen species (ROS) levels were reported to be significantly elevated in patients with RA [17].

Drug repurposing provides a cost-effective strategy for identifying new uses and for established new treatment for certain disease conditions with known safety profiles [18, 19]. Nitazoxanide, a broad-spectrum antiprotozoal with a well-documented safety profile [20], has been shown to inhibit STAT-3, JAK-2, and TLR-4/IL-1β signaling pathways [21–24].

Depression is a common RA-related comorbidity, which can also worsen disease outcomes [25]. Escitalopram, a selective serotonin reuptake inhibitor (SSRI), is commonly used for treating depression and has demonstrated anti-inflammatory and antioxidant properties particularly through suppressing pro-inflammatory cytokines and inhibiting endosomal TLR-4 signaling in RA tissues [26–28].

Based on the above-mentioned knowledge, we run this study to investigate the therapeutic efficacy and safety of nitazoxanide and escitalopram as adjuvant therapies in patients with RA, focusing on their effects on the STAT-3/JAK-2 and TLR-4/IL-1β signaling pathways, as well as oxidative stress modulation.

## Participants and methods

### Study design, setting, and participant selection

This randomized controlled parallel study was conducted on Egyptian patients with RA. The patients were diagnosed with RA according to the American College of Rheumatology/European League against Rheumatism criteria 2010 (ACR/EULAR 2010 criteria) [29]. The study was performed in accordance with the ethical standards declaration of Helsinki in 1964 and its later amendments. The institutional Research Ethics Committee of Mansoura University approved the study protocol (Approval Code #: TP/RE/06/22 ph-002). The clinical study was registered on “ClinicalTrials.gov” with an identifier number (NCT05480878) in July 29, 2022. All participants provided their written informed consent. Ninety patients were recruited from the outpatient clinic of the Physical Medicine, Rheumatology, and Rehabilitation Department at Mansoura University Hospital, Mansoura Governorate, Egypt, between August 2022 and July 2024. Computer generated code was used to randomize participants in a 1:1:1 ratio according to the consolidation standards of reporting trials (CONSORT) guidelines into one of the following groups: Group 1 (control group; *n* = 30) was treated with traditional therapy, which included oral or systemic corticosteroids, non-steroidal anti-inflammatory drugs (NSAIDS), and conventional disease modifying anti-rheumatic drugs (DMARDs), of which methotrexate, leflunomide, hydroxychloroquine, and sulfasalazine were implicated. Group 2 (Nitazoxanide group; *n* = 30) was treated with traditional therapy plus a daily oral dose of 1 gm nitazoxanide for 3 months (Nanozoxid®, Utopia Pharmaceuticals, Cairo, Egypt). Group 3 (Escitalopram group; *n* = 30) received traditional therapy plus a daily oral dose of 10 mg escitalopram for 3 months (Escitaloborg®, Borg Pharmaceuticals Industries, Alexandria, Egypt). Patients with active RA disease (the 28-joint disease activity score “DAS28” > 3.2) and aged between 18 and 75 years old were included in the study. We excluded patients with hypersensitivity to the study medications, pregnant and lactating women, patients with liver disease and renal impairment, patients with other inflammatory and autoimmune diseases, patients treated with TNF-α or IL-1β antagonists, patients with conditions associated with oxidative stress, and patients taking antioxidants.

### Demographic data

Demographic data were reported for all patients with RA including age, weight, and height measurement with subsequent calculation of body mass index (BMI) according to the formula: (BMI) = [Weight (kg) ÷ Height^2^ (m)]). In addition, data concern disease duration, gender, smoking habit, other health problems, medication history, and concurrent medications were collected as shown in Table 1.

**Table 1:** Demographic characteristics data of the study population

Variables		Groups						ANOVA
		Group 1 (Control) *N* = 30		Group 2 (Nitazoxanide) *N* = 30		Group 3 (Escitalopram) *N* = 30		*P*-value
Age (years)	Mean ± SD	49.67±	9.41	52.27±	8.06	49.067±	9.64	0.352
Weight (kg)	Mean ± SD	78.00±	2.95	78.83±	4.36	78.23±	3.48	0.660
Height (m)	Mean ± SD	1.69±	0.05	1.69±	0.05	1.70±	0.06	0.610
BMI (kg/m^2^)	Mean ± SD	26.99±	1.68	27.65±	2.53	27.05±	1.88	0.405
Disease duration (years)	Mean ± SD	6.07±	2.59	5.67±	2.84	5.87±	2.43	0.841
Chi-square		*N*	%	*N*	%	*N*	%	*P*-value
Gender	Male	4	13.33	2	6.67	2	6.67	0.578
	Female	26	86.67	28	93.33	28	93.33	
Smoking	Yes	7	23.33	10	33.33	5	16.67	0.319
	No	23	76.67	20	66.67	25	83.33	
Other health problems	Hypertension	9	30.00	8	26.67	8	26.67	0.946
	Diabetes	2	6.67	0	0.00	4	13.33	0.117
	Osteoarthritis	4	13.33	3	10.00	4	13.33	0.902
Traditional therapy	NSAID	30	100.00	30	100.00	30	100.00	-
	Corticosteroids	30	100.00	30	100.00	30	100.00	-
	Methotrexate	20	66.67	20	66.67	20	66.67	1.000
	Leflunomide	20	66.67	20	66.67	16	53.33	0.469
	Hydroxychloroquine	20	66.67	16	53.33	22	73.33	0.257
	Sulfasalazine	4	13.33	8	26.67	6	20.00	0.435

Data are presented as mean ± SD for continuous data, or numbers (percentages) for categorical data. Group 1 (Control): patients with RA received traditional treatment; Group 2 (Nitazoxanide): patients with RA received traditional therapy plus Nitazoxanide 1 gm/day for 3 months; Group 3 (Escitalopram): patients with RA received traditional therapy plus Escitalopram 10 mg/day for 3 months. Differences between groups were tested for significance using ANOVA for quantitative data and chi-square test for categorical data (*P* < 0.05). *P* < 0.05 is considered significant*NSAIDS* non-steroidal anti-inflammatory drugs

### Patient assessment

Patient assessments were conducted at the start of the trial and 3 months later as follows:

#### Clinical assessment

A rheumatologist performed a thorough and complete clinical evaluation of each patient. Medical history was reported to assert no interfering or interacting diseases or drugs. All patients were subject to local assessment to determine tender and swollen joint counts. Disease activity status was assessed using DAS28-CRP according to the following formula: DAS28-CRP = [0.56*sqrt (tender joint count) + 0.28 * sqrt (swollen joint count) + 0.36 * ln (CRP + 1)] * 1.10 + 1.15 [30] where high disease activity ≥ 5.1, low disease activity ≤ 3.2, and remission < 2.6.

#### Functional assessment

The validated Arabic Multidimensional Health Assessment Questionnaire (MDHAQ) [31] was used to evaluate the patients’ quality of life (QOL), which comprises the following: The Health Assessment Questionnaire-Disability Index (HAQ-DI) physical and physiological function score was calculated in the standard manner: The 14 items were rated on a scale of 0 to 3, where 0 represents no difficulty, 1 represents considerable difficulty, 2 represents severe difficulty, and 3 represents inability to do. The overall physical function score was calculated as the mean of the sum of the responses. It also included three visual analogue scales (VAS) (range 0–10) to evaluate pain, fatigue, and overall Health status. A 10-cm scale was employed, where 0 score represents no symptoms and a score of 10 represents very severe symptoms. The duration of morning stiffness (MS) in minutes was also assessed.

### Blood sample collection and biochemical assessment

A total of 10 mL of venous blood was withdrawn from each patient using sterile venipuncture, without frothing, and after minimal venous stasis using disposable syringes at baseline and 3 months after intervention. About 2 mL of blood was added into a vacutainer tube that contained potassium-ethylene-diaminetetraacetic acid (K-EDTA) for the assessment of hemoglobin level and for the determination of complete blood count (Sysmex®XN-1000TM; Automated HematologyAnalyzer Code: 19,732, Japan). The remaining 8 mL of the collected blood samples were centrifuged at 3000 rpm, and the sera were separated and divided into two portions. The first portion of the sera was used to rapidly measure serum creatinine (S.Cr) and serum blood urea nitrogen (BUN), which were measured using a fully automated Beckman Coulter/Olympus AU680 Chemistry Analyzer, Japan. The second portion of sera was kept frozen at (− 80 °C) until the assessment of the biological markers. Double antibody sandwich enzyme linked immune-sorbent assay (ELISA) kits were used for the assessment of serum level of hs-CRP (Sun Red, Biological technology Co. Ltd, Shanghai, China; Cat# 201–12-1806), STAT-3 (Sun Red, Biological technology Co. Ltd, Shanghai, China; Cat# 201–12-0651), JAK-2 (Sun Red, Biological technology Co. Ltd, Shanghai, China; Cat# 201–12-8695), TLR-4 (Sun Red, Biological technology Co. Ltd, Shanghai, China; Cat# 201–12-0347), interleukin-1β “IL-1β” (Sun Red, Biological technology Co. Ltd, Shanghai, China; Cat# 201–12-0144B), and MDA (Sun Red, Biological technology Co. Ltd, Shanghai, China; Cat # 201–12-1372).

### Assessment of patients’ adherence and drug’ safety

Patients were followed-up through weekly telephone calls and through monthly direct meetings during clinic visits to assess their compliance and to report any medication-related adverse reactions using an adverse drug reaction reporting form. Patients’ adherence to the study medication was evaluated by counting the pills and through the medication refilling rate. The Beck Depression Inventory Questionnaire was used to assess the impact of the study medication on depression.

### Primary and secondary outcomes

The study’s primary outcome was the change in DAS28-CRP score, HAQ-DI score, pain score, fatigue score, and PGA. The secondary outcome was the change in the biological markers (STAT-3, JAK-2, TLR-4, IL-1β, MDA).

### Sample size calculation

 The sample size was calculated based on Steven K. Thompson equation [32]:

$$n=\frac{N\times p\left(1-p\right)}{\left[\left[N-1\times\left(d^2\div z^2\right)\right]+p\left(1-p\right)\right]}$$

where *N* is the assumed population size (100,000,000), *d* is the alpha error proportion (0.05), *Z* is the *z* score (1.96) at 95% confidence level, and *P* is the population probability value (0.063) at 10% margin of error. Therefore, 30 participants were required for each treatment group.

### Statistical analysis

Data analysis was performed using version 24.0 of the IBM-SPSS statistical program (SPSS Inc., USA, 2016). The normality of the data was evaluated using Kolmogorov–Smirnov test. Chi-square test was used for statistical analysis of nominal data. Paired *t*-test was implicated to assess any significant difference within each group (baseline versus after treatment data). One-way analysis of variance (ANOVA) test was used to determine any Significant difference between the three study groups at baseline and 3 months after treatment, followed by Tukey’s HSD for multiple pairwise comparisons. Correlation between the measured variables was performed using Pearson correlation analysis. Data were presented as mean ± standard deviation, number, ratio, and percent. Significance level was set at *P*-value < 0.05.

## Results

**Fig. 1:**
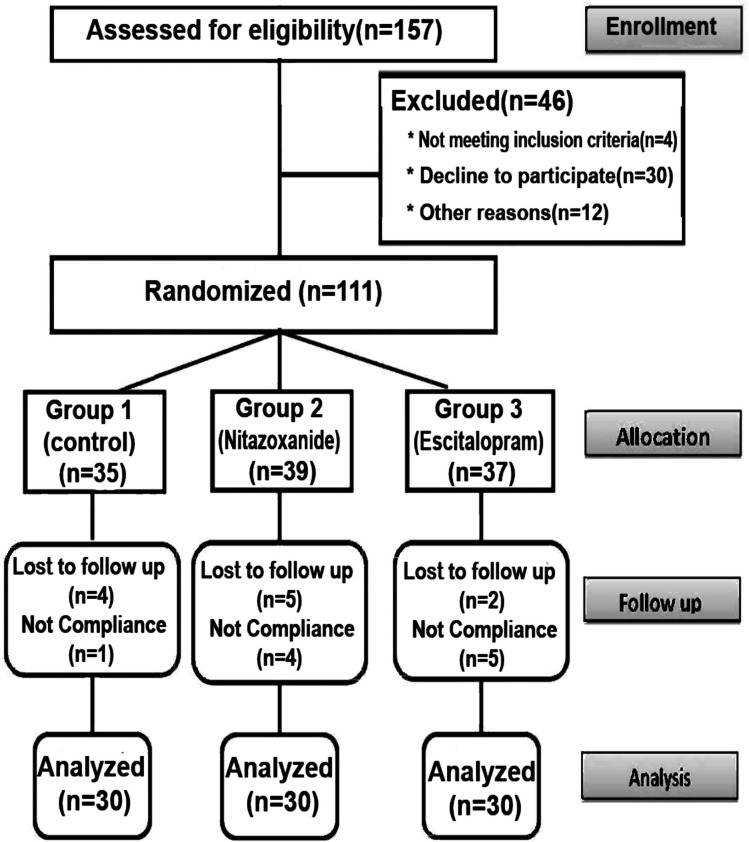
Flow- chart illustrating the participant’s enrollment and randomization

Figure 1 illustrates the participant flowchart. Out of 157 patients with RA screened for eligibility, 46 patients were excluded secondary to (lupus arthritis or juvenile RA), or declined to participate, or for other reasons (renal or hepatic disease, pregnancy, or lactation). Therefore, the remaining 111 patients were randomized into the three study groups. During the follow-up period, 21 patients were dropped out of the three study groups secondary to loss of follow-up or non-adherence. The final analysis included 90 participants, with 30 patients in each group.

### Demographic characteristics and other baseline data

The three study groups were statistically similar regarding their demographic data (*P* > 0.05), as shown in Table 1. The clinical, functional, and laboratory features for all participants in the three study groups at baseline showed non-significant difference (*P* > 0.05) as illustrated in Table 2.

**Table 2:** Selected clinical, functional and laboratory features for the three study groups at baseline

Variables	Group 1 (Control) *N* = 30	Group 2 (Nitazoxanide) *N* = 30	Group 3 (Escitalopram) *N* = 30	*P*-value
DAS28-CRP (score)	5.07 ± 1.17	5.39 ± 1.29	5.32 ± 1.32	0.590
HAQ-DI (score)	1.34 ± 0.41	1.51 ± 0.31	1.37 ± 0.25	0.121
MS (min)	30.00 ± 21.58	40.67 ± 21.32	34.00 ± 20.06	0.145
Pain Score (0–100 mm, VAS)	7.57 ± 1.61	8.43 ± 1.07	7.97 ± 1.54	0.069
Fatigue score (0–100 mm, VAS)	7.53 ± 1.72	7.63 ± 1.43	8.03 ± 1.54	0.428
PGA (0–100 mm, VAS)	7.60 ± 1.63	7.60 ± 2.01	7.77 ± 2.01	0.925
Hb (g/dL)	11.09 ± 1.37	11.21 ± 0.95	10.90 ± 1.23	0.595
WBCs (103/µL)	6.21 ± 1.96	6.43 ± 1.60	7.14 ± 1.65	0.105
Lymphocytes (103/µL)	2.13 ± 0.66	2.50 ± 0.55	2.40 ± 0.45	0.103
Platelets (10^3^/µL)	289.93 ± 54.68	292.33 ± 64.35	288.47.23 ± 60.55	0.969
BUN (mg/dL)	18.07 ± 3.55	17.43 ± 1.97	17.20 ± 2.66	0.467
S.cr (mg/dL)	0.87 ± 0.23	0.87 ± 0.25	0.83 ± 0.19	0.718
hs-CRP (mg/L)	46.71 ± 26.44	56.22 ± 21.49	58.71 ± 25.26	0.141
STAT-3 (pg/mL)	20.02 ± 6.35	23.50 ± 7.40	21.83 ± 7.03	0.158
JAK-2 (ng/mL)	0.56 ± 0.16	0.51 ± 0.21	0.53 ± 0.21	0.629
TLR-4 (ng/mL)	0.43 ± 0.17	0.41 ± 0.14	0.49 ± 0.18	0.140
IL-1β (pg/mL)	5.42 ± 1.43	5.58 ± 1.59	6.25 ± 1.19	0.059
MDA (nmol/mL)	8.20 ± 1.43	8.21 ± 3.66	10.25 ± 5.61	0.074

Data are presented as mean ± SD. Group 1 (Control): patients with RA received traditional treatment; Group 2 (Nitazoxanide): patients with RA received traditional therapy plus Nitazoxanide 1 gm/day for 3 months; Group 3 (Escitalopram): patients with RA received traditional therapy plus Escitalopram 10 mg/day for 3 months. *P* < 0.05 is considered significant*DAS28-CRP* disease activity score using C- reactive protein, *HAQ-DI* health assessment questionnaire-disability index, *MS* morning stiffness, *PGA* patient’s global assessment, *Hb* hemoglobin, *WBCs* white blood cells, *BUN* blood urea nitrogen, *S.cr* serum creatinine, *hs-CRP* high sensitivity C-reactive protein, *STAT-3* signal transducer and activator of transcription3, *JAK-2* janus kinase2, *TLR-4* toll-like receptors 4, *IL-1β* interleukin-1 beta, *MDA* malondialdehyde

### Summary of the main results

Three months after treatment and as compared to control group, the nitazoxanide group showed a significant decline in PGA (*P* = 0.042), pain score (*P* = 0.011), and serum levels of STAT-3 (*P* < 0.001), JAK-2 (*P* < 0.001), TLR-4 (*P* < 0.001), and IL-1β (*P* < 0.001). On the other hand, the escitalopram group produced a significant decrease in DAS28-CRP score (*P* = 0.029), HAQ-DI score (*P* = 0.001), pain score (*P* = 0.002), fatigue score (*P* = 0.003), and serum levels of JAK-2 (*P* = 0.001), TLR-4 (*P* < 0.001), IL-1β (*P* < 0.001), and MDA (*P* < 0.001) with a non-significant variation in STAT-3 serum level (*P* = 0.586). As compared to nitazoxanide group, the escitalopram group produced a significant decline in fatigue score (*P* < 0.001) and serum levels of both IL-1β (*P* = 0.023) and MDA (*P* < 0.001).

### Effect of interventions on anthropometric, clinical, and functional assessments

The change in anthropometric, clinical, and functional outcomes 3 months after treatment in all study groups is shown in Table 3. Regarding anthropometric assessment, 3 months after treatment, there was non-significant variation *n* BMI for all study groups compared to baseline values or among the three study groups (*P* > 0.05).

**Table 3:** Change in anthropometric, clinical, and functional assessments outcomes after 3 months of treatment in all study groups

Variable	Group 1 (Control) *N* = 30		(P) paired *t*-test	Group 2 (Nitazoxanide) *N* = 30		(P) paired *t*-test	Group 3 (Escitalopram) *N* = 30		(P) paired *t*-test
	Before	After		Before	After		Before	After	
BMI (kg/m^2^)	31.61 ± 6.62	31.80 ± 6.82	0.240	32.83 ± 6.62	33.01 ± 6.62	0.261	31.12 ± 7.32	31.32 ± 7.23	0.298
DAS28-CRP (score)	5.07 ± 1.17	5.21 ± 1.32	0.439	5.39 ± 1.29	4.44 ± 1.23*	< 0.001	5.32 ± 1.32	4.38 ± 1.17*^#^	< 0.001
HAQ-DI (score)	1.34 ± 0.41	1.44 ± 0.44	0.070	1.51 ± 0.31	1.25 ± 0.41*	< 0.001	1.37 ± 0.25	1.07 ± 0.31*^#^	< 0.001
MS (min)	30.00 ± 21.58	31.17 ± 22.15	0.804	40.67 ± 21.32	20.83 ± 17.52*	< 0.001	34.00 ± 20.06	20.33 ± 13.45*^#^	< 0.001
Pain score (0–100 mm, VAS)	7.57 ± 1.61	7.77 ± 2.13	0.586	8.43 ± 1.07	6.23 ± 1.99*^#^	< 0.001	7.97 ± 1.54	5.93 ± 1.89*^#^	< 0.001
Fatigue score (0–100 mm, VAS)	7.53 ± 1.72	7.70 ± 1.91	0.326	7.63 ± 1.43	8.03 ± 1.97	0.103	8.03 ± 1.54	6.10 ± 1.58*^# $^	< 0.001
PGA (0–100 mm, VAS)	7.60 ± 1.63	7.77 ± 1.74	0.096	7.60 ± 2.01	6.63 ± 1.83*^#^	< 0.001	7.77 ± 2.01	6.87 ± 1.79*	0.001

Data are presented as mean ± SD. Group 1 (Control): patients with RA received traditional treatment; Group 2 (Nitazoxanide): patients with RA received traditional therapy plus Nitazoxanide 1 gm/day for 3 months; Group3 (Escitalopram): patients with RA received traditional therapy plus Escitalopram 10 mg/day for 3 months. *P* < 0.05 is considered significant*BMI* body mass index, *DAS28-CRP* disease activity score using C-reactive protein, *HAQ-DI* health assessment questionnaire-disability index, *MS* morning stiffness, *PGA* patient’s global assessment^*^Significant compared to baseline values (paired *t*-test) (*P* < 0.05)^#^Significant change compared to group 1 (ANOVA/post hoc test) (*P* < 0.05)^$^Significant change compared to group 2 (ANOVA/post hoc test) (*P* < 0.05)

Regarding clinical and functional assessments, the data obtained with the current study revealed that, 3 months after intervention and as compared to baseline data, the control group (group 1) showed non-significant changes in DAS28-CRP score, HAQ-DI score, MS duration (min), pain score, fatigue score, and PGA as compared to baseline data (*P* > 0.05) as shown in Table 3. Three months after intervention and as compared to baseline data, the nitazoxanide group (group 2) showed significant improvement in DAS28-CRP score (*P* < 0.001), HAQ-DI score (*P* < 0.001), MS duration (*P* < 0.001), pain score (*P* < 0.001), and PGA (*P* < 0.001). However, the nitazoxanide group (group 2) showed non-significant change in fatigue score (*P* = 0.103) as shown in Table 3. Regarding the escitalopram group (group 3), the comparison of data obtained 3 months after intervention with the baseline data revealed that this group produced significant improvements in DAS28-CRP score (*P* < 0.001), HAQ-DI score (*P* < 0.001), MS duration (*P* < 0.001), pain score (*P* < 0.001), fatigue score (*P* < 0.001), and PGA (*P* = 0.001), as shown in Table 3.

Three months after treatment, the comparison of the three study groups showed that the nitazoxanide group (group 2) produced a significant decline in PGA (*P* = 0.042) and pain score (*P* = 0.011) as compared to the control group. On the other hand, and as compared to the control group, the escitalopram group (group 3) showed a significant decrease in DAS28-CRP score (*P* = 0.029), HAQ-DI score (*P* = 0.001), MS duration (*P* = 0.048), pain score (*P* = 0.002), and fatigue score (*P* = 0.003). Additionally, the escitalopram group showed a significant decline in fatigue score compared to the nitazoxanide group (*P* < 0.001), as postulated in Table 3.

### Effect of interventions on assessed biological markers

Regarding the effect of intervention on the assessed biological markers, the data obtained with this study demonstrated that, 3 months after intervention and as compared to baseline data, the control group (group 1) showed non-significant variations in all measured variables (*P* > 0.05). Three months after intervention and as compared to baseline data, the nitazoxanide group (group 2) produced a significant decrease in the serum level of hs-CRP (*P* < 0.001), STAT-3 (*P* < 0.001), and JAK-2 (*P* < 0.001), TLR-4 (*P* < 0.001), and IL-1β (*P* < 0.001), which was associated with a non-significant change in the MDA serum level (*P* = 0.197). As regarding the escitalopram group and as compared to baseline data, 3 months after treatment this group showed a statistically significant decrease in the serum levels of hs-CRP (*P* < 0.001), TLR-4 (*P* < 0.001), IL-1β (*P* < 0.001), and MDA (*P* < 0.001), which was associated with non-significant variations in the serum levels of STAT-3 (*P* = 0.307) and JAK-2 (*P* = 0.060) as demonstrated in Table 4.

**Table 4:** Effect of interventions on assessed biological markers among RA patients

Variable	Group 1 (Control) *N* = 30		(P) paired *t*-test^*^	Group 2 (Nitazoxanide) *N* = 30		(P) paired *t*-test	Group 3 (Escitalopram) *N* = 30		(P) paired *t*-test
	Before	After		Before	After		Before	After	
hs-CRP (mg/mL)	46.71 ± 26.44	53.18 ± 21.19	0.198	56.22 ± 21.49	37.81 ± 17.84*^#^	< 0.001	58.71 ± 25.26	39.27 ± 17.19*^#^	< 0.001
STAT-3 (pg/mL)	20.02 ± 6.35	22.83 ± 6.48	0.063	23.50 ± 7.40	12.49 ± 5.60*#	< 0.001	21.83 ± 7.03	21.12 ± 7.84	0.307
JAK-2 (ng/mL)	0.56 ± 0.16	0.59 ± 0.15	0.166	0.51 ± 0.21	0.28 ± 0.13*^#^	< 0.001	0.53 ± 0.21	0.44 ± 0.18	0.060
TLR-4 (ng/mL)	0.43 ± 0.17	0.43 ± 0.17	0.586	0.41 ± 0.14	0.29 ± 0.10*^#^	< 0.001	0.49 ± 0.18	0.25 ± 0.08*^#^	< 0.001
IL-1β (pg/mL)	5.42 ± 1.43	6.23 ± 1.57	0.428	5.58 ± 1.59	3.81 ± 1.01*^#^	< 0.001	6.25 ± 1.19	2.98 ± 0.92*^# $^	< 0.001
MDA (nmol/mL)	8.20 ± 1.43	8.28 ± 1.82	0.658	8.21 ± 3.66	8.12 ± 3.72	0.197	10.25 ± 5.61	4.83 ± 2.51*^# $^	< 0.001

Data are presented as mean ± SD. Group 1 (Control): patients with RA received traditional treatment, Group 2 (Nitazoxanide): patients with RA received traditional therapy plus Nitazoxanide 1 gm/day for 3 months, Group 3 (Escitalopram): patients with RA received traditional therapy plus Escitalopram 10 mg/day for 3 months. *P* < 0.05 is considered significant*hs- CRP* high sensitivity C-reactive protein, *STAT-3* signal transducer and activator of transcription3, *JAK-2* Janus kinase 2, *TLR-4* toll-like receptors4, *IL-1β* interleukin-1 beta, *MDA* malondialdehyde^*^Significant compared to baseline values (paired t-test) (*P* < 0.05)^#^Significant change compared to group 1 (ANOVA/post hoc test) (*P* < 0.05)^$^Significant change compared to group 2 (ANOVA/post hoc test) (*P* < 0.05)

The comparison of the three study groups revealed that, 3 months after treatment and as compared to the control group (group 1), the nitazoxanide group (group 2) showed a significant decrease in the serum levels of STAT-3 (*P* < 0.001), JAK-2 (*P* < 0.001), TLR-4 (*P* < 0.001), and IL-1β (*P* < 0.001). On the other hand, 3 months after treatment and as compared to the control group (group 1), the escitalopram group (group 3) showed a significant decrease in the serum level of JAK-2 (*P* = 0.001), TLR-4 (*P* < 0.001), IL-1β (*P* < 0.001) and MDA (*P* < 0.001), which was associated with a non-significant change in STAT-3 serum level (*P* = 0.586), as shown in Table 4.

The comparison between the nitazoxanide group (group 2) and the escitalopram group (group 3) 3 months after treatment demonstrated that the escitalopram group produced a significant decline in the serum level of IL-1β (*P* = 0.023) and MDA (*P* < 0.001) as compared with the nitazoxanide group, as postulated in Table 4.

### Correlation among measured serum biomarkers

**Fig. 2:**
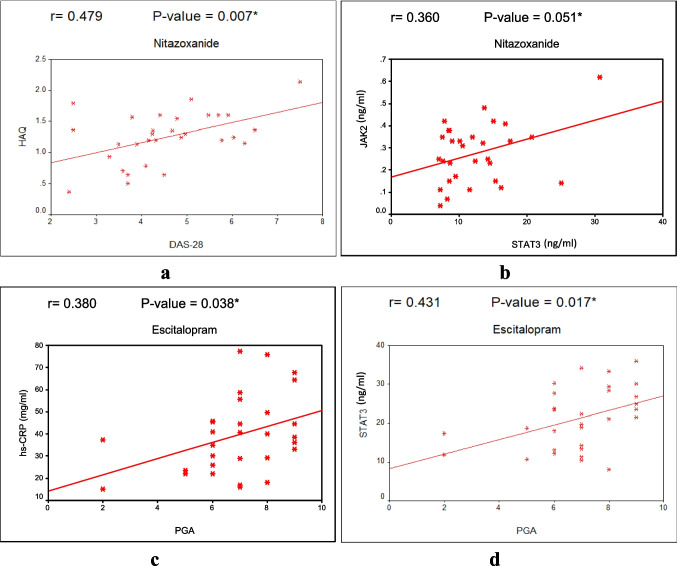
The Pearson correlati[F0341] on analysis between the measured variables. **a** Positive correlation between HAQ-DI and DAS28-CRP in Group 2 (Nitazoxanide). **b** Positive correlation between JAK-2 and STAT-3 in Group2 (Nitazoxanide). **c** Positive correlation between PGA and hs-CRP in Group3 (Escitalopram). **d** Positive correlation between PGA and STAT-3 in Group3 (Escitalopram) [F0341]LE: Please check if all figure captions are presented/captured correctly

Figure 2 illustrates the Pearson correlation analysis between the measured variables, which revealed a significant positive correlation in the nitazoxanide group between HAQ-DI and DAS28-CRP (*r* = 0.479, *P* = 0.007) and JAK 2 with STAT-3 (*r* = 0.360, *P* = 0.051). Regarding the escitalopram group, PGA showed a significant positive correlation with both hs-CRP (*r* = 0.380, *P* = 0.038) and STAT-3 (*r* = 0.431, *P* = 0.017).

### Safety and tolerability of the study medications

In order to document any potential adverse effects associated with the study medications, patients were monitored during the study period via phone calls and direct meeting during clinic visits. Concerning drug-related complications, no severe adverse reactions were observed with anyone of the study participants. Most of the reported adverse effects were minor, controllable, and temporary, which appeared at the initial period of the treatment and disappeared with time since the participants became tolerant to the study medications Chromatouria was the only significant nitazoxanide related adverse effect (*P* < 0.001). Other reported side effects, such as headache and increased appetite were statistically non-significant. Regarding escitalopram, it was noted that this drug significantly decreased the incidence of depression (*P* = 0.021). Headache and anorexia were found to be statistically non-significant side effects for escitalopram (*P* > 0.05), as shown in Table 5.

**Table 5:** Reported side effects among the three study groups

Side-effect	Group 1 (Control) *N* = 30	Group 2 (Nitazoxanide) *N* = 30	Group 3 (Escitalopram) *N* = 30	*P*-value
Chromaturia	0 (0%)	9 (30%)	0 (0%)	< 0.001*
Headache	9 (30%)	10 (33.33%)	11 (36.67%)	0.861
GIT disturbance	5 (16.67%)	4 (13.33%)	3 (10%)	0.749
Increased depression	10 (33.33%)	10 (33.33%)	2 (6.67%)	0.021*
Cough	4 (13.33%)	2 (6.67%)	1 (3.33%)	0.338
Increased appetite	0 (0%)	2 (6.67%)	0 (0%)	0.129
Anorexia and insomnia	2 (6.67%)	1 (3.33%)	0 (0%)	0.355

Data presented as number and percent. Group 1 (Control): patients with RA received traditional treatment; Group 2 (Nitazoxanide): patients with RA received traditional therapy plus Nitazoxanide 1 gm/day for 3 months; Group 3 (Escitalopram): patients with RA received traditional therapy plus Escitalopram 10 mg/day for 3 months. *P* < 0.05 is considered significant^*^Significant variation compared to other groups (chi-square test) (*P* < 0.05)Figure (1): Flow- chart illustrating the participant’s enrollment and randomizationFigure (2): The Pearson correlation analysis between the measured variables. **a** Positive correlation between HAQ-DI and DAS28-CRP in Group 2 (Nitazoxanide). b Positive correlation between JAK-2 and STAT-3 in Group2 (Nitazoxanide). c Positive correlation between PGA and hs-CRP in Group3 (Escitalopram). d Positive correlation between PGA and STAT-3 in Group3 (Escitalopram)

## Discussion

Rheumatoid arthritis (RA) is an autoimmune condition with a complicated origin [1]. One approach for drug research is drug repurposing, which aims at repurposing already-approved drugs for novel purposes [18, 19]. According to the best of the authors’ knowledge, this study is the first one aimed at evaluating and comparing the effects of nitazoxanide and escitalopram on clinical and laboratory parameters related to inflammation and angiogenesis in patients with RA. The new trend in this study is the implication of nitazoxanide and escitalopram as anti-inflammatory and immunomodulatory adjuvant therapy for patients with RA, through their suggested interruption of the STAT-3/JAK-2 signaling pathway, TLR-4/IL-1β signaling pathway, and their ability to modulate oxidative stress.

During the current study, the three research groups were statistically Similar at baseline; therefore, any changes in the measured outcomes after treatment are attributed to study medications and surely are not related to individual variation. More than 75% of the study participants were female, which seems consistent with a former concept demonstrated that RA disease is more common in women [2].

According to the findings obtained with the current study, patients with RA who received nitazoxanide and escitalopram as adjuvant therapies demonstrated significant improvements in their clinical and functional status, as evidenced by improvements in their DAS28-CRP score, HAQ-DI score, MS duration, and PGA. This improvement is confirmed by the presence of significant correlation among these scores in both groups and by the noted correlation between these scores with some of the measured serum biomarkers (hs-CRP and STAT-3), notably in the escitalopram group. These correlations seem logical since the inflammatory state is well-known to decrease quality of life. These findings illustrate that the study medications have clinical effects in addition to their impact on the assessed biological biomarkers.

The STAT-3/JAK-2 signaling pathway plays a pivotal role in immune regulation [33, 34]. Interestingly, STAT-3/JAK-2 overexpression is closely associated with angiogenesis and inflammation, and the activation of STAT-3 was reported to be correlated with poor prognosis in many inflammatory diseases [5–8]. Therefore, the STAT-3/JAK-2 signaling pathway could be an attractive target for the treatment of patients with RA [35]. Our research findings showed that, as compared to baseline data, only the nitazoxanide-treated group produced significant reductions in STAT-3 and JAK-2 serum levels. This finding comes in the same line with some former studies concluded that nitazoxanide is a potent inhibitor of the transcription factor STAT-3 and its potential anticancer effects are sustained by blocking STAT3-dependent gene expression [36, 37]. Another study reported that nifuroxazide inhibits tyrosine phosphorylation of the kinase JAK-2 [23]. Furthermore, these beneficial effects of nitazoxanide are compatible with a previous preclinical study, which deduced that nifuroxazide mitigates angiogenesis in Ehrlich’s solid carcinoma via inhibition of JAK-2/STAT-3 signaling [38]. Our result seems also in parallel with an in vitro study, which was conducted on an acetic acid-induced ulcerative colitis model in rats and proved the inhibitory effect of nifuroxazide on STAT3/JAK2 signaling [39]. Furthermore, our result is in agreement with former findings that identified a nephron-protective effect of nifuroxazide in diabetic kidney, through its effective inhibition of STAT-3 activation, and also with other former studies conducted on various types of cancer, which reported the efficacy of nifuroxazide to inhibit cancer growth and to counteract metastasis via inhibition of STAT-3 phosphorylation [39–42]. In this context, the ability of nitazoxanide’s to block STAT-3/JAK-2 signaling offers a new approach for the management of several inflammatory conditions since the anti-inflammatory action of such drugs stems from its ability to interrupt the STAT-3/JAK-2 signaling pathway, which in turn makes it an attractive candidate for the treatment of patients with RA.

On the other hand, data addressing the immunomodulatory characteristic of selective serotonin reuptake inhibitors (SSRIs) and their effects on cytokine production are well established [43–45]. Although SSRIs are believed to possess anti-inflammatory activity, escitalopram failed to interrupt the STAT-3/JAK-2 signaling pathway during the current study. To the best of our knowledge, there are no available data about the effect of escitalopram on the STAT-3/JAK-2 signaling pathway in patients with RA, and there are still considerable gaps in understanding the mechanistic characterization of the immunomodulatory effects of escitalopram. Inconsistent with our finding, Taler et al. stated that the SSRI paroxetine suppressed signal transducer and activator of transcription-3 (STAT-3) protein expression in human peripheral blood mononuclear cells [46]. Some investigators stated that paroxetine modulates immune responses by activating a JAK-2/STAT-3 signaling pathway [47]. In this context, the data addressing paroxetine effects on the JAK-2/STAT-3 signaling pathway seem controversial. These conflicting data may be related to the fact that paroxetine, a SSRI with high affinity to serotonin receptor (5‐HT1 receptor), may exert its immune-modulatory effect via serotonin [48]. This aforementioned notion could be inferred from a former finding that demonstrated that the 5‐HT1 receptor is responsible for paroxetine JAK-2/STAT-3 signaling modulation. Additionally, JAK-2 is regarded as a downstream Signaling pathway for the 5-HT1 receptor [49], and paroxetine may exert its anti-inflammatory effects via 5-HT systems in immune cells [48]. All previously mentioned data could explain the lack of direct effect of escitalopram on JAK-2/STAT-3 and relate the immunomodulatory effect of escitalopram to its action on 5-HT receptors and serotonin. Additionally, during the current study, STAT-3 showed a significant positive correlation with both JAK-2 and PGA, which may indicate a correlation between angiogenesis and inflammation. Our former finding about this link is consistent with some earlier studies [50, 51].

Additionally, the goal of the current study was to assess how nitazoxanide and escitalopram affected TLR-4 and IL-1ß signaling. The TLR-4 signal pathway is crucial for immunological response and RA [52]. As previously deduced, the oligomerization domain-like receptors containing protein-3 (NLRP-3) inflammasome and nucleotide-binding are activated by TLR-4 signaling with subsequent release of IL-1β [53]. The inflammatory response is significantly influenced by IL-1β, which has a vital role in a number of cellular processes, such as cell division, proliferation, and apoptosis [24]. During the current study, nitazoxanide significantly decreased TLR-4 and IL-1β serum levels. The effect of nitazoxanide on TLR-4, IL-1β serum levels seems to be matched with a previously reported in vitro study postulated that nitazoxanide suppressed the production of IL-1β, IL-6, and TNF-α from lipopolysaccharide-stimulated macrophages and decreased cytokine gene transcription [54]. Our result also comes in accordance with a previous clinical study, which was conducted by Khodir et al. on patients with COVID-19 and demonstrated the potential role of nifuroxazide in attenuating sepsis-induced systemic inflammation and multiple organ failure through interruption of TLR-4/inflammasome NLRP3/IL-1β signaling [24]. On the other hand, the significant favorable effect of escitalopram on TLR-4 and IL-1β comes in consonance with a recently reported study that postulated that treatment with escitalopram selectively inhibits endosomal TLR signaling and suppresses inflammatory cytokine production in human and murine models of RA, which provides a potential mechanism for its anti-inflammatory action [26]. Furthermore, our result comes in parallel with some former studies that proved that SSRIs interrupt TLR signaling and suppress inflammatory cytokine production [43–47].

In fact, the production of lipid peroxides and reactive oxygen species (ROS) due to disease activity may be a significant factor in the pathogenesis of RA. Furthermore, oxidative stress is believed to promote angiogenesis by increasing growth factor receptor-2 (VEGFR2) auto-phosphorylation and stimulating VEGF expression [15, 16]. Moreover, elevated serum levels of circulating MDA in those with RA indicate decreased antioxidant capacity and increased oxidative stress and can predict endothelial dysfunction. According to the best of our knowledge, there are no available data about the effect of nitazoxanide on MDA serum level in patients with RA. The data obtained with the current study demonstrated that nitazoxanide did not produce any significant beneficial effect on serum MDA level. In contrast to our findings, other authors reported that nitazoxanide had an antioxidant effect by reducing oxidative stress and mitophagy flow through ROS-mediated mitophagy initiation and lysosomal dysfunction in bladder cancer [55]. Furthermore, Khodir et al. demonstrated that nifuroxazide administration significantly improved lung and heart oxidative status [24]. These contradictory findings may be attributed to the insufficient nitazoxanide dose, which is incapable of exerting receptor-independent antioxidant activity, which would inhibit the formation of free radicals and attenuate chemotaxis. This aforementioned information could be explained by the results of a previously published research, which showed that the antioxidant activity of nitazoxanide is maintained with large dosages of nitazoxanide (200 mg/kg) taken once daily for 3 weeks [55]. Furthermore, these conflicting findings might be explained by the differences in the study duration, the nature of the illness, and the differences between researches conducted on humans and animals [56]. On the other hand, our findings revealed that the serum MDA level decreased significantly only in the group treated with escitalopram. According to this former finding, escitalopram may have antioxidant properties and reduce oxidative stress in patients with RA. Our previous finding is consistent with other studies hypothesized that citalopram has antioxidant property and contributes to the improvement of endothelial function in RA and other conditions [27, 28]. Additionally, the presence of a positive correlation between MDA levels and IL-1ß indicates the link between oxidative stress and IL-1ß release. This former finding is in parallel with former studies, which showed that oxidative stress and inflammation are inter-related and oxidative stress can influence cytokine production and signaling [27, 28].

The follow-up period of the current study was 3 months, which seems acceptable and comes in consonance with some previous studies, which demonstrated that a 3-month follow-up period is sufficient to assess the disease activity [57–60].

Regarding safety and tolerability of the study medications, nitazoxanide showed a good tolerability and safety profile during the current study. Chromaturia was the only significant nitazoxanide-related adverse effect. The data obtained with the current study concerning the safety of nitazoxanide seem in matching with many former studies demonstrated a low incidence of adverse effects with the implication of nitazoxanide [61, 62]. Escitalopram significantly decreased the Beck Depression Inventory Questionnaire score and subsequently improved depressive symptoms in patients with RA. Depression is still a significant comorbidity in patients with RA, and it is believed to increase their risk of disability and death [25]. Furthermore, some data point to the biological correlation between mood disorder and the substrates that cause inflammation [25]. These aforementioned data could justify and support the implication of escitalopram for patients with RA. Our finding regarding the safety of escitalopram comes in accordance with the notion that escitalopram is a relatively old medication with well-known safety [63].

It is worth mentioning that according to the British National Formulary, nitazoxanide and escitalopram were not reported to interact with the other medications used during the current study [23]. In this context, the overall results of the current study offer a novel opportunity and therapeutic venue for counteracting RA through the implication of nitazoxanide and escitalopram as adjuvant therapies to the conventional therapy.

The points of strength of the current study include its design as a randomized controlled parallel study and its priority, according to the best of the authors’ knowledge, as the first clinical study aimed at evaluating the efficacy and safety of nitazoxanide and escitalopram as adjuvant therapies for patients with RA. However, the current study has some limitations, including the relatively small sample size, the short follow-up period, and being an open-label single center study. Therefore, further large-scale, more longitudinal and multi-center studies are still needed to confirm the promising results obtained with this study.

## Conclusion

At the end of this randomized controlled parallel study, both nitazoxanide and escitalopram produced significant improvements in functional and clinical assessments in patients with rheumatoid arthritis. Both drugs exerted a beneficial effect on the serum levels of the biological markers involved in the pathogenesis of rheumatoid arthritis. Nitazoxanide was superior to escitalopram in reducing STAT-3 and JAK-2 serum levels, while escitalopram was superior to nitazoxanide in attenuating oxidative stress. Both drugs were safe and well-tolerated. In this context, we assume that nitazoxanide and escitalopram could be implicated as adjuvant therapies for the treatment of patients with rheumatoid arthritis. However, further large-scale and more longitudinal studies are still recommended.

## Data Availability

Availability of data and materials: Data are available upon reasonable request from the corresponding author.
